# A review of artificial intelligence-assisted omics techniques in plant defense: current trends and future directions

**DOI:** 10.3389/fpls.2024.1292054

**Published:** 2024-03-05

**Authors:** Sneha Murmu, Dipro Sinha, Himanshushekhar Chaurasia, Soumya Sharma, Ritwika Das, Girish Kumar Jha, Sunil Archak

**Affiliations:** ^1^ Indian Agricultural Statistics Research Institute, Indian Council of Agricultural Research (ICAR), New Delhi, India; ^2^ Central Institute for Research on Cotton Technology, Indian Council of Agricultural Research (ICAR), Mumbai, India; ^3^ National Bureau of Plant Genetic Resources, Indian Council of Agricultural Research (ICAR), New Delhi, India

**Keywords:** artificial intelligence, abiotic stress, biotic stress, machine learning, deep learning, plants

## Abstract

Plants intricately deploy defense systems to counter diverse biotic and abiotic stresses. Omics technologies, spanning genomics, transcriptomics, proteomics, and metabolomics, have revolutionized the exploration of plant defense mechanisms, unraveling molecular intricacies in response to various stressors. However, the complexity and scale of omics data necessitate sophisticated analytical tools for meaningful insights. This review delves into the application of artificial intelligence algorithms, particularly machine learning and deep learning, as promising approaches for deciphering complex omics data in plant defense research. The overview encompasses key omics techniques and addresses the challenges and limitations inherent in current AI-assisted omics approaches. Moreover, it contemplates potential future directions in this dynamic field. In summary, AI-assisted omics techniques present a robust toolkit, enabling a profound understanding of the molecular foundations of plant defense and paving the way for more effective crop protection strategies amidst climate change and emerging diseases.

## 1 Introduction

Plant defense against both biotic (living organisms like pathogens and pests) and abiotic (environmental factors such as drought, salinity, and extreme temperatures) stress is of paramount importance in ensuring global food security. With the world’s population steadily growing, the demand for crops is increasing, making efficient plant protection strategies a critical need. The ability to safeguard crops from the ravages of diseases, pests, and adverse environmental conditions is essential to maintain agricultural productivity and secure the global food supply. The substantial impact of these stressors is underscored by the extensive economic losses, as evidenced by the multi-billion-dollar reductions in crop yields (FAO 2017 report).

Biotic stressors, such as pathogens and pests, pose a constant threat to crop health. Pathogens, including fungi, bacteria, and viruses, can devastate entire plant populations, leading to substantial economic losses and food shortages. Likewise, pests, ranging from insects to nematodes, have the potential to decimate crops, leading to decreased yields and quality.

Food crops around the world face substantial yield reductions due to microbial diseases and pest infestations. These losses are significant, with rice experiencing an average loss of 30.3%, maize at 22.6%, wheat at 21.5%, soybeans at 21.4%, and potatoes at 17.2% (Savary et al., 2019). Plant diseases can be especially devastating, leading to yield reductions of up to 50% in specific regions, particularly impacting small-scale farmers and posing substantial economic challenges. Additionally, plant diseases negatively influence species diversity, increase the costs associated with disease control measures, and even have repercussions on human health (Ristaino et al., 2021). The emergence of new plant diseases and pest outbreaks carries substantial economic implications for agriculture, posing threats to food security, national stability, and public health (Anderson et al., 2004). In the coming years, it is expected that the changing distribution of pathogens due to climate variations and increased global trade will result in a higher prevalence and greater severity of emerging plant diseases (Bebber et al., 2013). A notable recent example is the outbreak of coffee rust, caused by *Hemileia vastatrix*, in Central America, which led to significant crop losses and economic crises (Avelino et al., 2015).

On the other hand, abiotic stress factors are non-living elements that challenge plant growth and survival. These include prolonged droughts, extreme temperatures, soil salinity, and heavy metal contamination. The impacts of abiotic stressors are often subtle and insidious, affecting crop yields, nutritional content, and overall plant health. Efforts to combat both biotic and abiotic stressors have traditionally relied on a combination of methods, including conventional breeding, chemical treatments, and agronomic practices. However, these approaches are often reactive and may not provide effective protection, especially in the face of emerging pathogens or rapidly changing environmental conditions.

Recently, advanced omics techniques, have revolutionized the exploration of molecular-level stress mechanisms in plants (Shen et al., 2022). These methods provide extensive information, revealing intricate networks involving genes, proteins, and metabolites during plant defense against biotic stress. However, the substantial data generated by these omics technologies poses a significant challenge in terms of analysis and interpretation, necessitating the development of highly effective computational tools.

Artificial Intelligence (AI) has emerged as a potent instrument for unraveling vast omics datasets and understanding intricate mechanisms underlying plant responses to stress. These techniques offer a deeper understanding of the genetic, molecular, physiological, and phenotypic aspects of plant defense, enabling the development of novel strategies to bolster crop resilience and mitigate stress-induced damage. In traditional plant defense research, the complex networks of genes, proteins, and metabolites involved in stress responses posed challenges due to the high volume and complexity of biological data. However, the advent of AI-assisted omics techniques has ushered in innovative solutions to tackle and interpret this vast data landscape. These techniques encompass diverse AI methodologies, efficiently unraveling and modeling the intricate relationships between molecular components and stress responses.

In this review, we intend to provide an overview of different omics studies involving various stress factors in plants. As we navigate through the intersection of AI and plant omics, we explore cutting-edge developments in the realm of plant defense against both biotic and abiotic stressors and confront its challenges. This review critically explores the advantages of AI over traditional methods, delves into the challenges of AI in plant omics, and future directions in plant defense research, highlighting the potential for sustainable agricultural practices that enhance crop protection, stress tolerance, and global food security.

## 2 Different omics in plant defense research

Plant defense research encompasses various “omics” technologies, each offering unique insights into the molecular mechanisms underlying plant responses to pathogens and environmental stresses. In the following sections, we’ll provide a brief overview of the primary types of data each omics approach can provide when applied to research on plant stress.

### 2.1 Genomics

Plants, as stationary organisms, have evolved sophisticated defense mechanisms against various stressors, including biotic and abiotic factors. The combination of genomics and AI has become a potent tool for unraveling the genetic foundations of plant defense. This review explores the current and future applications of AI-assisted genomics in understanding both biotic and abiotic stress responses in plants. It covers the identification of resistance genes, characterization of defense pathways, and improvement of stress tolerance. The challenges, ethical considerations, and potential breakthroughs in this evolving field are also discussed.

Genome-wide association studies (GWAS) play a crucial role in genomic strategies to enhance crop resilience against abiotic stress. Mangin et al. (2017) illustrate the significance of GWAS in evaluating abiotic stress impacts on sunflower oil content. Previous studies identified Quantitative Trait Loci (QTLs) associated with maize yield under heat and water stress (Millet et al., 2016). Environmental variables in GWAS investigations revealed Single Nucleotide Polymorphisms (SNPs) linked to sorghum drought stress, with 213 genomic regions associated with drought tolerance (Lasky et al., 2015; Spindel et al., 2018).

Epigenetics, involving heritable modifications beyond DNA sequences, combines with genomics in the emerging field of epigenomics. This integration unveils genetic regulation in cellular responses to stress, with epigenomic processes responding to environmental conditions and stressors. Genome-level investigations are necessary to scrutinize these phenomena across developmental stages or assess deviations due to plant diseases (Callinan and Feinberg, 2006; Muthamilarasan et al., 2019). The revolutionary CRISPR-Cas9 technology, originating from bacteria as a defense mechanism against viruses, has transformed genome editing. CRISPR-Cas systems extensively edit eukaryotic genomes, providing opportunities to engineer crop plants for enhanced resilience against both abiotic and biotic stresses (Kumar and Jain, 2015).

### 2.2 Transcriptomics

Transcriptomics, the study of an organism’s complete set of RNA transcripts within specific cells or tissues (transcriptome), is a dynamic field with potential for analyzing gene expression responses to various stimuli over defined timeframes (Raza et al., 2021). Transcriptome profiling, the approach used in this field, allows the investigation of gene expression differences, providing insights into the functions of specific genes.

In various crops like sorghum and rice, transcriptome studies have identified gene sets with altered expression in response to stressors such as drought, heat, osmotic stress, and hormonal treatments (Dugas et al., 2011; Jin et al., 2013; Johnson et al., 2014). These analyses are crucial for understanding gene expression changes during growth and stress responses, offering valuable insights for functional studies. Transcriptomics has proven significant in unraveling stress responses and developmental processes in crops, as demonstrated in RNA-seq studies in foxtail millet and sweet potato, revealing tissue-specific gene expression responses to abiotic and biotic stress (Li et al., 2017). The application of RNA-seq in rice, maize, and rapeseed oil research has aided in identifying genes responsive to drought stress (Bhardwaj et al., 2015). Comparative transcriptomic analysis enables exploration of distinct gene expression profiles across diverse crop species facing stress, identifying shared genes and revealing intricate cross-talk pathways (Li et al., 2013; Zhu et al., 2013). These findings emphasize the significance of regulatory networks governing stress tolerance genes, offering potential for enhancing crop traits through genetic improvement.

### 2.3 Proteomics

Proteomics, as a comprehensive approach to studying proteins, plays a crucial role in understanding how plants respond to both biotic and abiotic stresses. The four main aspects of proteomics—sequence, structural, functional, and expression proteomics—offer a holistic view of the complex interactions within plant cells (Aizat and Hassan, 2018). In sequence proteomics, scientists identify amino acid sequences using advanced techniques like high-performance liquid chromatography, providing insights into the building blocks of proteins (Twyman et al., 2013). Structural proteomics focuses on understanding the three-dimensional structures and functions of proteins, employing various methods like computer-based modeling, NMR, crystallization, electron microscopy, and X-ray diffraction (Woolfson et al., 2018). Functional proteomics delves into the roles of proteins, employing methodologies such as Y2H assays and protein microarray profiling to decipher the specific functions of different proteins within the cellular context. Quantitative proteomics, exemplified by the iTRAQ method, allows researchers to measure changes in protein expression levels in response to stresses, providing valuable information about how plants react to environmental challenges (Liu et al., 2015; Zhu et al., 2018; Yang et al., 2020).

In the context of plant responses to biotic stress, proteomics has proven pivotal. Studies involving Vitis species and other crops showcase the ability of proteomic analyses to identify stress-responsive proteins and uncover translational modifications like phosphorylation and ubiquitination. This information aids in understanding the intricate molecular dynamics underlying plant defense mechanisms and pathogen virulence (Mosa et al., 2017). Similarly, in the realm of abiotic stress, such as drought, proteomics reveals proteins associated with stress response in crops like wheat. The integration of proteomics and phosphoproteomics explores diverse functions in response to various stressors, contributing to the identification of both resistant and susceptible crop cultivars against these challenges. Additionally, the combination of proteomics with other omics disciplines like metabolomics and functional genomics enhances our understanding of stress biology, facilitating the identification of molecular markers for breeding programs (Margaria et al., 2013; Yang et al., 2013; Zhang et al., 2014). The various proteomic techniques, including LC-MS/MS, MALDI-TOF, SDS-PAGE, and iTRAQ, are extensively applied in different crops to investigate their responses to both biotic and abiotic stress conditions (Mohammadi et al., 2012; Ramalingam et al., 2015). The insights gained from proteomic studies significantly contribute to unraveling the molecular mechanisms by which plants adapt to environmental challenges, ultimately leading to advancements in crop yield improvement and stress resilience.

### 2.4 Metabolomics

Metabolomics, a study of metabolites in biological systems, is crucial for understanding the plant metabolome and revealing regulatory mechanisms under stress conditions. This field, integrated with next-generation sequencing, provides insights into molecular responses in crops, offering a broader perspective on biochemical processes influencing gene functionality.

In plant defense against stress and pathogens, metabolites play a vital role, identified through gas chromatography-mass spectrometry as biomarkers in rice varieties facing the GMB1 pathogen. Similar strategies reveal metabolite accumulation in response to other pathogens in rice and barley, showcasing the importance of metabolomics in understanding plant responses to biotic stress.

Wheat crops also exhibit the presence of phenylpropanoid and phenolic metabolites in response to biotic stress. Metabolomics is particularly vital in plant systems due to the abundant production of metabolites. Secondary metabolites like polyamines, identified in rice crops under drought stress, highlight the relevance of environmental metabolomics in understanding plant responses to abiotic stresses. Various metabolomics techniques, including LC/GC-MS, GC/EI-TOF-MS, HPLC, and NMR, have been widely employed in crops like rice, tomato, maize, and soybean, providing valuable insights into their responses to both abiotic and biotic stress conditions.

### 2.5 Phenomics

Plant phenomics involves systematically acquiring and analyzing multi-dimensional traits across various crop growth stages, from cellular to field levels. This process relies on a three-step approach: trait identification, data conversion into quantifiable measurements, and computational methodologies for analysis. High-throughput phenotyping platforms are crucial in the initial phase, while computational strategies, particularly machine learning (ML) algorithms, play a pivotal role in subsequent stages. The performance of crop phenotypes is intricately linked to genetic factors and environmental conditions. The continuous evolution of sensors, imaging technologies, and analytical methodologies has led to the development of numerous dedicated infrastructure platforms for phenotyping.

Abiotic stresses such as drought, salinity, and nutrient deficiencies pose significant challenges to crop production, eliciting complex plant responses. Phenotyping for stress resistance is imperative for breeding resilient crops. Drought stress, marked by reduced water availability, can be evaluated using ground-based platforms equipped with thermometer sensors and RGB cameras. Unmanned Aerial Vehicles (UAVs) integrated with thermal cameras facilitate quicker scanning of larger plots for identifying drought-resistant genotypes. Salinity stress, impacting stomatal conductance, is observed through visible to near-infrared spectral reflectance images. Scanalyzer3D aids in characterizing salinity tolerance mechanisms. Image-based methods, encompassing RGB and fluorescence imaging, assess tissue ion concentrations to gauge salinity tolerance. Hyperspectral imaging, coupled with ML, predicts traits associated with salinity stress. Crop nutrient deficiencies, especially nitrogen, affect chlorophyll content, growth, and disease susceptibility, with monitoring conducted through sensors like RGB, multispectral, and hyperspectral sensors. Mobile platforms incorporating these sensors estimate nitrogen content efficiently. Agriculture confronts threats from diseases and pests, and the integration of resistance genes presents a cost-effective strategy. Biotic stress induces changes in various plant characteristics, and advanced phenotyping platforms utilizing optical sensors effectively detect and manage biotic stress factors in crops.

## 3 Basics of AI techniques

AI involves creating computer systems to perform tasks associated with human intelligence, such as learning, problem-solving, and decision-making. ML, a subset of AI, focuses on developing algorithms and statistical models that enable computers to perform tasks without explicit programming. DL, a specialized field within ML, involves training artificial neural networks to mimic the human brain’s structure, utilizing deep architectures with multiple layers for automatic hierarchical feature extraction.

**Figure 1:**
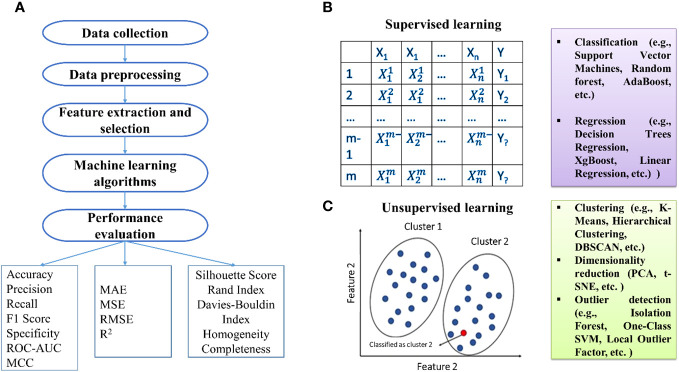
**(A)** Basic steps involved in the development of machine learning models. Types of machine learning: **(B)** supervised and **(C)** unsupervised techniques.

The initial phase of ML includes data collection, especially in sequencing data like RNA sequencing studies (**Figure 1**). Denoising methods enhance expression recovery. Supervised ML uses diverse features for training data representation, including amino acid sequence information and physicochemical properties (Sperschneider et al., 2018). Feature selection is crucial, and methods fall into three categories: filter, wrapper, and embedding methods (Guyon and Elisseeff, 2006; Guyon et al., 2008; Khalid et al., 2014).

Algorithm selection is pivotal, and ML algorithms can be categorized into supervised, semi-supervised, and unsupervised. Supervised methods establish relationships between input factors and outcomes based on training examples. Unsupervised methods, primarily clustering, identify data patterns without relying on known outcomes. Practical algorithms include SVM, DT, RF, ANN, and NB for supervised learning, while k-means, independent component analysis, and hierarchical clustering are used for unsupervised learning. Semi-supervised learning handles input data with both labeled and unlabeled information, with examples like the label propagation algorithm.

### 3.1 Unsupervised ML

Unsupervised ML methods fulfill two primary functions: clustering, which groups data based on similarity, and dimension reduction, generating representative features from numerous variables. A widely utilized clustering method is k-means, aiming to create non-overlapping clusters of observations. Principal Component Analysis (PCA) is a common technique for dimension reduction, transforming high-dimensional observations into a smaller set of uncorrelated principal components (PCs) to simplify subsequent analyses.

### 3.2 Supervised ML

Support Vector Machine (SVM) is a supervised learning method created by Vapnik (1999) for binary classification tasks. It operates in an n-dimensional space, forming a hyperplane to maximize the margin between distinct data classes. The choice of kernel functions, such as linear or nonlinear options like polynomial or radial basis, significantly influences its performance.

The k-nearest-neighbor (KNN) algorithm, introduced by Altman (1992), is another supervised learning technique that classifies data points by identifying the ‘k’ nearest neighbors with known labels. The classification is based on a majority vote among these neighbors. While user-friendly, traditional KNN methods may have longer computation times (Borah et al., 2020).

The Decision Tree (DT) classifier, developed by Quinlan (1986), follows a branch-test approach. It recursively partitions data based on attributes until a specified stopping condition is met, creating a tree-like structure. The classification path can be traced from the root node to each leaf node (Schietgat et al., 2010).

Random Forest (RF), introduced by Breiman in 2001, is an ensemble algorithm utilizing a group of DTs to achieve a consensus on accurate classification. Classification trees are constructed by randomly selecting from training datasets, and the predictions from each tree are combined to provide an overall prediction for each observation (Breiman, 2001).

### 3.3 Major deep learning architectures

ML has gained widespread popularity, and DL methods, particularly associated with Artificial Neural Network (ANN) architectures, are gaining increasing attention. DL autonomously learns from raw input data, capturing intricate patterns without extensive domain expertise. Unlike traditional ML, which relies on discrete or continuous output predictions based on data counts or measurements, DL excels in direct feature learning from input datasets, eliminating the need for conventional feature engineering (Bonetta and Valentino, 2020).

ANNs, dating back to McCulloch and Pitts (1943), mimic biological neurons and feature a learning process facilitated by synaptic connections. Deep Neural Networks (DNNs) encompass multiple hidden layers, effectively forming a complex structure. Recurrent Neural Networks (RNNs), introduced by Sperduti and Starita (1997), are suitable for supervised learning with feedback loops for cyclic data processing. Convolutional Neural Networks (CNNs), introduced by Lecun et al. (1998), excel in identifying relevant features without human supervision, while Graph Convolutional Networks (GCNs), introduced by Schaffer (1989), handle intricate problems through complex architectures (Zhou et al., 2022).

Transformers, rooted in a self-attention mechanism, find application in natural language processing tasks like text translation, improving task parallelization (Vaswani et al., 2017). Ensemble classifiers enhance decision-making by combining outputs from different models, introduced by Dietterich (2000). Clustering-based methods, like the k-means algorithm, provide an unsupervised approach for predicting protein functions by exploiting direct and indirect interactions (Hou, 2017; Yan and Wang, 2022).

Each algorithm, including ML and DL models, offers unique capabilities catering to the complexities of omics datasets. Understanding their distinctive strengths and weaknesses is crucial before exploring their applications. **Table 1** provides a concise overview summarizing the key attributes defining their performance.

**Table 1:** Strengths and weaknesses of different AI-methods.

Method	Pros	Cons	Task
k-nearest-neighbor	▪ Simple implementation for handling multi-class problems. ▪ Consistency in selected hyperparameters. ▪ Utilizes a non-parametric algorithm.	▪ Slower processing speed. ▪ More suitable for datasets with a limited number of input variables. ▪ Inefficiency with imbalanced data. ▪ Prone to sensitivity issues with outliers.	▪ Classification ▪ Regression
Decision Tree	▪ Easy data pre-processing without the need for scaling or normalization. ▪ Capable of handling both numerical and categorical features effectively. ▪ Facilitates a clear visual representation of output.	▪ Longer training times, contributing to slower processing. ▪ Prone to overfitting issues. ▪ For complex or large datasets, may provide inadequate predictions. ▪ Not ideally suited for datasets which are not balanced.	▪ Classification ▪ Regression
Random Forest	▪ Capable of adapting to dynamic neural networks. ▪ Computational power suitable for handling non-linear systems.	▪ Problem of exploding and vanishing gradient for long sequences. ▪ Training process tends to be slow and complex.	▪ Classification ▪ Regression
Support Vector Machine	▪ Efficient when dealing with distinctly separable classes. ▪ Well-suited for two-class classification tasks. ▪ Effective for high dimensional datasets. ▪ Relatively memory-efficient.	▪ For noisy and large datasets, it may not be suitable. ▪ Requires preprocessing of data. ▪ Prone to the risk of overfitting. ▪ Involves computationally costly processes. ▪ Output has low interpretability.	▪ Classification ▪ Regression
Ensemble Classifier	▪ Achieves higher predictive accuracy compared to individual models. ▪ Capable of handling both linear and non-linear data. ▪ Reduces overfitting and bias. ▪ Produces more stable and reduced noisy predictions.	▪ Computationally expensive. ▪ Interpretability is limited. ▪ Memory intensive.	▪ Classification ▪ Regression ▪ Clustering
Graphical Neural Network	▪ Consistency in parameter use during the training iteration. ▪ Cost-effective data storage. ▪ Adaptive learning of the importance of neighbours in a graph-based system.	▪ Algorithm’s processes can be untraceable. ▪ High computational costs.	▪ Classification ▪ Regression ▪ Clustering
Clustering Model	▪ Efficient and requires fewer computations. ▪ Intuitive without the need for pre-set clusters. ▪ Identifies outliers without predetermined clusters. ▪ Offers more flexibility compared to k-means. ▪ Insensitivity to the choice of distance metric, allowing for hierarchy visualization.	▪ Inconsistency in dealing with outliers. ▪ Challenges in selecting an optimal number of clusters. ▪ For high dimension datasets, there may be inconsistency in performance. ▪ Utilizes all data points that are available. ▪ Lower efficiency ▪ Increased time complexity.	▪ Clustering

### 3.4 Validation strategies

ML predictions in plant genomics research can be validated through a variety of methods. Xavier (2021) emphasized the importance of cross-validation in comparing different algorithms for genomic prediction. K-fold cross-validation, a widely used method, involves randomly dividing training samples into k subsets, reserving one for validation, and using the others for training (Sun et al., 2020). Evaluation metrics, derived from the confusion matrix, include sensitivity, specificity, accuracy, precision, F1-score, and Matthews correlation coefficient (MCC). Sensitivity gauges correctly predicted positives, specificity assesses correctly predicted negatives, accuracy reflects overall correct predictions, and precision measures correctly predicted positives among TP and FP. The F1-score combines precision and recall, while MCC is valuable for imbalanced datasets (Jiao and Du, 2016). The receiver operating characteristic (ROC) curve, evaluated with false positive rate (FPR) and true positive rate (TPR), and the area under the ROC curve (AUC) serve as performance measures, with higher AUC indicating superior predictor performance (Xu-Hui et al., 2009).

Individual-based models demand a context-oriented approach due to their complex and variable interaction structure (Leye et al., 2009; Kubicek et al., 2015). This approach involves separately assessing different model levels and employing various techniques, including visual inspection, statistical comparison, expert involvement, and experimental validation. In the realm of engineering and scientific models, a proposed statistical validation approach links validation experiments to the target application and considers the importance of measurements (Hills and Leslie, 2003). When applied context-dependently, these strategies enhance the support for hypotheses generated by the model. Maron et al. (2014) underscored the necessity of experiments exploring the role of abiotic factors in plant-animal interactions. Wang et al. (2018) introduced pattern-oriented modeling as an effective means to verify and validate functional-structural plant models, showcasing its predictive capabilities in plant growth. Abele et al. (2013) introduced an ontology-based approach to validate plant models, ensuring their accuracy through Semantic Web reasoning. Bylesjö et al. (2007) discussed the O2PLS method for integrating transcript and metabolite data in plant biology, providing a means to validate and interpret models. Together, these studies emphasize the significance of experimental and computational validation strategies in plant omics research.

For ML models applied to plant omic data, context specificity is crucial, as highlighted by Isewon et al. (2022), especially for improving agronomic traits and developing resilient crop varieties. Silva et al. (2019) further emphasizes the necessity of ML approaches in plant molecular biology, particularly in the analysis of pathogen-effector genes. Fukushima et al. (2009) underscores the importance of integrating multiple omics data, including metabolomics, to reconstruct complex networks in plant systems. Collectively, these studies support the use of context-specific ML models and the integration of omics data for a more comprehensive understanding of plant biology.

The effectiveness of ML prediction in plant stress omics research finds objective validation through various methods. John et al. (2022) compare classical and ML-based phenotype prediction methods, noting the varying performance of different models in real-world data. Ghosal et al. (2017) enhance the interpretability of ML models by applying a ML framework to identify and classify foliar stresses in soybean plants, isolating visual symptoms for each stress. Singh et al. (2018) emphasize the importance of standardizing visual assessments, deploying imaging techniques, and using ML tools for data assimilation and feature identification in plant stress phenotyping. Together, these studies underscore the potential of ML in accurately predicting and identifying plant stress, with a focus on interpretability and standardization.

## 4 Why choose machine learning for plant-omics data over traditional methods?

ML is increasingly preferred over traditional methods in plant-omics data analysis due to its adept handling of large, complex datasets. High-throughput sequencing technologies have ushered in a wealth of information, enabling biologists to explore intricate associations, decode stress responses in plants, and unravel complexities in genomic responses (Singh et al., 2016). However, challenges such as high dimensionality, uncertainty, and non-independence among variables in plant omics data have emerged. Traditional statistical models face limitations in handling this complexity (Altman and Krzywinski, 2018; Niazian and Niedbała, 2020).

ML, especially DL, has proven efficient in overcoming these challenges, providing accurate analyses of plant characteristics affected by genotype and environment interactions (Arsenovic et al., 2019). Unsupervised and semi-supervised ML algorithms have been applied to plant systems biology, facilitating big data analysis without the need for large labeled training sets (Yan and Wang, 2022). ML’s application extends to improving plant agronomic traits through the integration of large omics data (Isewon et al., 2022). Studies by Farooq et al. (2022), Isewon et al. (2022), and Silva et al. (2019) highlight the superiority of ML methods, particularly decision tree-based ensemble models (Gokalp and Tasci, 2019), in genomic prediction and integrative analysis of plant omics data. ML’s potential in deciphering complex interactions in plant molecular biology, including pathogen effector genes and plant immunity, is underscored (Silva et al., 2019).

In transcriptomics, ML methods stand out for enhancing the sensitivity of differential expression gene identification (Wang et al., 2018). However, the use of non-linear ML models in differential expression analysis may have limitations, leading to the recommendation of eXplainable Artificial Intelligence for model interpretation and gene set identification (Sabbatini and Calegari, 2023). The integration of ML with traditional biological information is emphasized for learning biological dynamics from large datasets, complementing traditional modeling approaches (Xu and Jackson, 2019; Gilpin et al., 2020). Various ML tools, including tree-based methods, Bayesian models, network-based fusion methods, kernel methods, matrix factorization models, and deep neural networks, play a crucial role in connecting multi-view biological data (Li et al., 2016).

ML’s proficiency in multivariate analysis is advantageous for considering numerous variables simultaneously, leading to the discovery of new biomarkers and predictive model development (Reel et al., 2021). Its application in improving agronomic traits in plant omics research is evident, although challenges in fully realizing the potential of integrating multiomics data remain, with scaling difficulties being a major obstacle (Noor et al., 2019). The complexity of high-dimensional omics data necessitates sophisticated methods for feature selection and information extraction. ML’s advantage lies in its ability to discern and prioritize the most relevant features, as demonstrated by Du et al. (2019) in the analysis of RNA-sequencing data related to salt stress response in rice. The use of ML-based feature selection methods, including principal component analysis and LASSO, effectively revealed submodules associated with observed traits.

ML excels in prediction and classification tasks, allowing researchers to forecast phenotypic outcomes and identify potential biomarkers. Its scalability is crucial for large-scale omics data, setting it apart from traditional methods facing computational challenges. In conclusion, the shift towards ML in plant omics research is driven by its unique strengths in addressing data intricacies, enabling predictive modeling, and facilitating an exploratory approach to data analysis.

Pattern recognition is another strength of ML, enabling the discovery of intricate patterns and associations within complex datasets. In omics research, where uncovering subtle patterns may provide novel insights into biological mechanisms, this capability is highly valuable. Moreover, ML is adaptable to the heterogeneity often observed in omics datasets due to biological variability and technical differences. Its flexibility and generality, with less reliance on assumptions about data distribution, make it suitable for various data types and experimental designs. The exploratory nature of ML, facilitating the uncovering of hidden patterns and relationships, is crucial in omics research. This aspect allows researchers to generate hypotheses and identify novel avenues for further investigation.

In the realm of multi-omics analysis, the primary objective is constructing Gene Regulatory Networks (GRNs). While ChIP-seq experiments for profiling Transcription Factors’ (TFs) binding sites are limited in plants, the inference of GRNs heavily relies on expression data (Bubb and Deal, 2020). Traditional correlation-based methods and the Mutual Information (MI) algorithm face challenges in distinguishing regulatory direction and considering temporal delays between gene expressions (Banf and Rhee, 2017; Redekar et al., 2017; Haque et al., 2019). To overcome these limitations, Probabilistic Graphical Models (PGM), such as GENIST and JRmGRN, have been introduced, though they require high spatiotemporal resolution in expression data (de Luis Balaguer et al., 2017; Deng et al., 2018).

ML has revolutionized the inference of GRNs, integrating multi-omics data to enhance accuracy (Walley et al., 2016; Ko and Brandizzi, 2020). iDREM (interactive dynamic regulatory events miner), employing a hidden Markov model, reconstructs temporal GRNs in response to biotic and abiotic stresses using transcriptomic, proteomic, and epigenomic datasets (Ding et al., 2018). With the emergence of single-cell RNA sequencing (scRNA-seq), tools like GRNBoost2, based on the GENIE3 framework, facilitate cell-specific GRN inference (Moerman et al., 2019). The SCENIC analytical pipeline, incorporating multiple tools, efficiently analyzes datasets within 2 hours comprising of 50,000 cells and 10,000 genes (Van de Sande et al., 2020).

In summary, the shift towards ML in plant omics research is driven by its unique strengths in addressing the intricacies of omics data, accommodating multiple variables, integrating diverse datasets, providing predictive modeling and classification capabilities, and facilitating an exploratory approach to data analysis.

## 5 Application of AI in plant omics against stress

**Figure 2:**
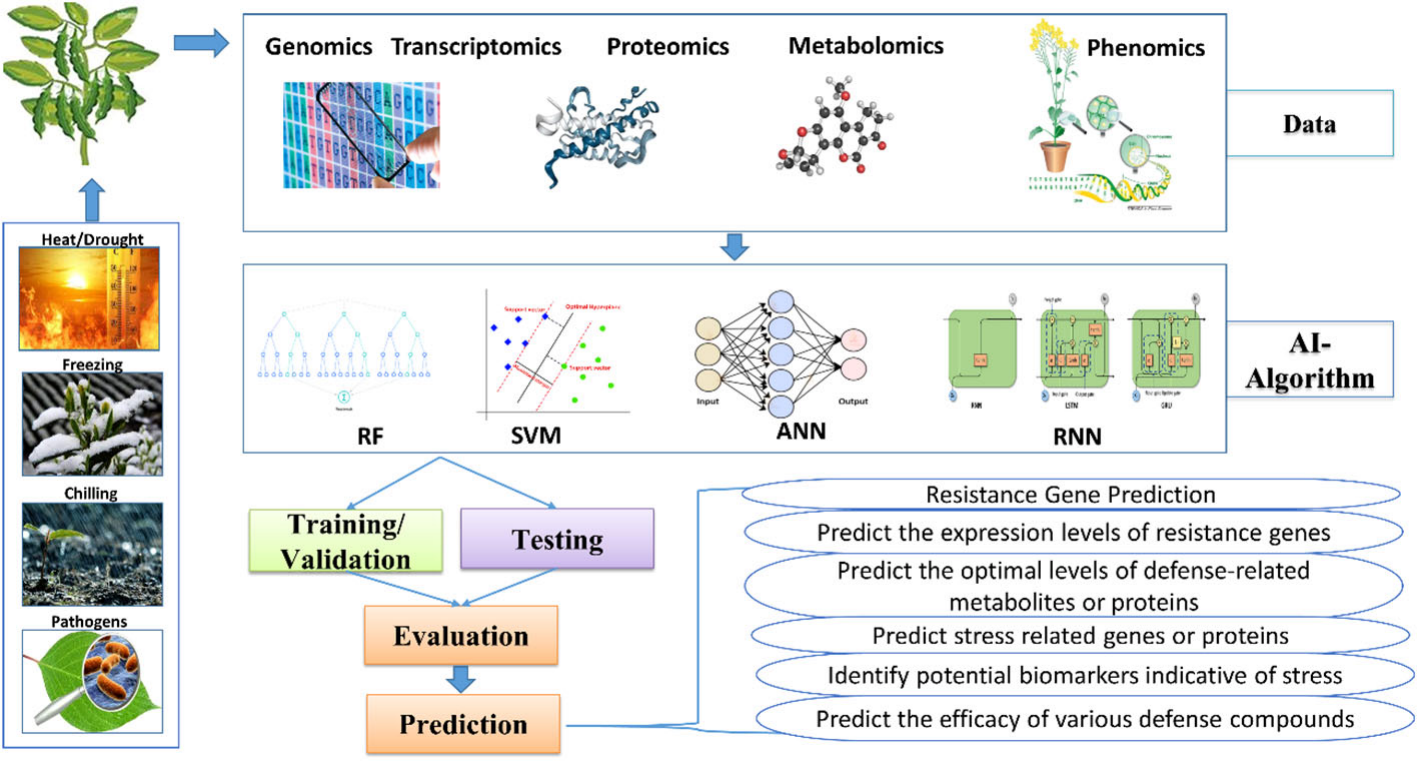
Different omics in plant defense research.

AI-assisted omics techniques in plant defense research represent a cutting-edge approach, combining advanced molecular technologies with AI for a deeper understanding of how plants respond to stresses (**Figure 2**). Traditional research faced challenges in deciphering complex gene, protein, and metabolite networks, but AI-supported omics methods provide innovative solutions. These techniques rapidly identify crucial components in defense pathways, discover biomarkers, and reveal hidden patterns, enhancing our comprehension of plant defense processes. Integrating multi-omics data sources offers a holistic understanding, and as AI techniques evolve, they hold promise for developing stress-resistant crops, optimizing agricultural practices, and ensuring sustainable food production (Arabnia and Tran, 2011; Yan and Wang, 2023).

### 5.1 AI-assisted genomics

Machine learning (ML) algorithms play a pivotal role in identifying stress resistance genes, aiding breeders and researchers in enhancing crop production. Liang et al. (2011) utilized a variant of the Support Vector Machine (SVM) algorithm to identify key genes associated with drought resistance in A. thaliana. Shikha et al. (2017) demonstrated the superior performance of Bayes algorithms, identifying critical SNPs for drought resistance in maize. ML algorithms have been applied beyond drought resistance, with Wang et al. (2013) using an SVM-based model to predict salt resistance genes in rice. Ravari et al. (2015) assessed salt tolerance in Iranian wheat genotypes, identifying effective indices for predicting salt-tolerant varieties using artificial neural network (ANN) analysis. Schwarz et al. (2020) employed ML techniques to explore the cis-regulatory code governing the response to iron deficiency in Arabidopsis roots.

In the realm of plant disease resistance, SVM and its variants are widely employed, as demonstrated by Pal et al. (2016), achieving high accuracy in predicting disease resistance proteins. ML has also been instrumental in predicting pathogen effector proteins, with Sperschneider et al. (2016) developing EFFECTORP, the first ML classifier for fungal effectors. Despite the focus on disease resistance genes, ML algorithms hold promise in understanding genes susceptible to plant diseases, contributing significantly to agricultural practices (Yang and Guo, 2017). The application of ML in exploring plant single-cell genomic data offers opportunities to unravel cellular heterogeneity, decode regulatory networks, and identify novel cell types. Recent studies (Silva et al., 2019; Raimundo et al., 2021) highlight ML’s potential in tasks such as generating low-dimensional representations, classifying cell types, inferring trajectories, deducing gene regulatory networks, and integrating multimodal data. Challenges related to low sequencing coverage and amplified artifacts in single-cell RNA (scRNA) sequencing are addressed by ML approaches, such as the SIMLR algorithm (Wang et al., 2017) and neural network models (Lin et al., 2017), providing more reliable insights into the intricate landscape of single-cell genomics. Despite these advancements, further research is needed to fully unlock the potential of ML in plant single-cell genomics. The current application of ML in identifying stress resistance genes is limited to a few plant species, urging the extension of ML utilization to other economically significant plants for a comprehensive understanding of stress resistance mechanisms and accelerated breeding efforts.

### 5.2 AI-assisted transcriptomics

In a comprehensive exploration of miRNAs and their roles in plant stress responses, Asefpour Vakilian (2020) conducted a study focusing on both biotic and abiotic stresses. The research employed feature selection algorithms to delve into the contributions of individual miRNAs in Arabidopsis thaliana responses to various abiotic stresses, including drought, salinity, cold, and heat. Utilizing information theory-based feature selection, key miRNAs, such as miRNA-169, miRNA-159, miRNA-396, and miRNA-393, were identified as significant contributors to the plant’s reactions to these stressors. The study harnessed regression models, including DT, SVMs, and NB, revealing the exceptional predictive capabilities of SVM with a Gaussian kernel, achieving a high coefficient of determination (R² = 0.96) for plant stress based on miRNA concentrations.

Contrary to the traditional belief in separate signaling pathways for abiotic and biotic stresses in plants, a study on rice by Shaik and Ramakrishna (2014) shed light on the intricate relationship between these stress responses. Through a meta-analysis of microarray studies, the researchers identified shared stress-responsive genes in rice, revealing conserved expression patterns across both types of stresses for approximately 70% of the common differentially expressed genes. Advanced data analysis techniques and ML models, including recursive-support vector machine and random forests decision tree, effectively distinguished between abiotic and biotic stress responses based on gene expression profiles. The recursive-support vector machine achieved a perfect 100% accuracy in classifying these stress types, identifying 196 genes that significantly contributed to the accurate classification.

In a study led by Meng et al. (2021), supervised classification models were employed to identify genes responding transcriptionally to cold stress. Surprisingly, models trained solely with features derived from genome assemblies displayed modest reductions in performance compared to those incorporating a wider range of data. Notably, models trained with data from one plant species demonstrated remarkable success in predicting gene responses to cold stress in related species, even when transferring predictions between cold-sensitive and cold-tolerant species. Multi-species models, trained using data from multiple species, outperformed single-species models when it came to cross-species prediction accuracy. This approach, driven by ML, shows promise in accelerating the understanding of gene expression responses to environmental stresses across diverse plant species.

In response to abiotic stress, such as heat or cold, plants undergo significant changes in gene expression to adapt and survive. In a study conducted by Zhou et al. (2022), transcriptome profiling of maize genotypes exposed to heat or cold stress revealed extensive alterations in transcript abundance. Motifs near the transcription start sites (TSSs) of genes responsive to thermal stress were found to be enriched. Predictive models developed using these motifs could forecast gene expression responses to stress, with enhanced accuracy focusing on motifs within unmethylated regions near the TSSs. However, challenges emerged when applying these models across different maize genotypes, indicating reduced performance when transferred between genotypes.

In a recent study, Pradhan et al. (2023a) employed artificial intelligence, specifically ML, to tackle the challenge of identifying long non-coding RNAs (lncRNAs) associated with abiotic stress responses in plants. Abiotic stresses significantly impact crop yields, emphasizing the importance of developing stress-resistant crop cultivars. The researchers devised a novel computational model capable of predicting abiotic stress-responsive lncRNAs. They utilized a dataset comprising stress-responsive and non-stress-responsive lncRNA sequences for binary classification. Various ML algorithms, including SVM, were applied, and the representation of lncRNAs was numeric based on Kmer features. Through effective feature selection strategies, the SVM model demonstrated impressive cross-validation accuracy at 68.84%. Further validating its robustness, the model exhibited an accuracy of 76.23% on an independent test dataset. To enhance accessibility, the researchers also introduced an online prediction tool called ASLncR.

In a parallel study, Pradhan et al. (2023a) directed their focus towards predicting microRNAs (miRNAs) associated with specific abiotic stresses, such as cold, drought, heat, and salt. Given the vital role of miRNAs in plant responses to these stresses, their identification holds significance for breeding stress-resistant crops. Leveraging ML, specifically SVM, the researchers developed a computational model for predicting stress-responsive miRNAs. They utilized pseudo-K-tuple nucleotide compositional features to numerically represent miRNAs. The SVM model achieved high cross-validation prediction accuracies ranging from 87.71% to 90.15% across different stress conditions. To facilitate the utility of this computational tool, an online prediction server named ASmiR was established.

Similarly, Meher et al. (2022a) contributed to the field by developing a ML-based method for predicting miRNAs responsive to abiotic stresses. They worked with three types of datasets: miRNA, pre-miRNA, and pre-miRNA + miRNA. Using pseudo-K-tuple nucleotide compositional features, sequence data was transformed into numeric feature vectors. SVM was employed for prediction, and the model achieved respectable results. The area under the receiver operating characteristics curve (auROC) and area under the precision-recall curve (auPRC) percentages ranged from 65.64% to 77.94%. Overall prediction accuracies for the independent test set ranged from 62.33% to 69.21%. To facilitate the application of this approach, the researchers provided an online prediction server named ASRmiRNA. The method shows promise in advancing the identification of abiotic stress-responsive pre-miRNAs and miRNAs.

### 5.3 AI-assisted proteomics

Meher et al. (2022a) employed computational methods and machine learning (ML) to streamline the identification of abiotic stress-responsive genes (SRGs) across various stress conditions, achieving accuracy levels of 60% to 78% with the SVM model. They introduced an online prediction application, ASRpro, for broader accessibility. In the realm of plant-pathogen protein-protein interactions (PPIs), Yang et al. (2019) utilized Random Forest to predict known plant-pathogen PPIs, showcasing enhanced accuracy by incorporating sequence data and network attributes. The InterSPPI web server was introduced to support ongoing research. Karan et al. (2023) focused on plant-microbe interactions, predicting PPIs for rice and blast fungus interactions with ML models achieving up to 95% accuracy on experimental datasets. The specificity of the model to *O. sativa* and *M. grisea* was confirmed through assessments against other pathogen-host datasets. Ahmed et al. (2023) introduced a novel activation function, Gaussian Error Linear Unit with Sigmoid (SIELU), in a deep learning model for classifying unknown abiotic stress protein sequences, outperforming other models with high accuracies ranging from 80.78% to 95.11%.

### 5.4 AI-assisted metabolomics

Liu et al. (2017) conducted a study focusing on the classification of 216 plants based on their incomplete metabolite content. Their research employed a network clustering algorithm to group metabolites with similar structures. Plants were represented as binary vectors, and hierarchical clustering was used for classification. Despite working with incomplete data, the approach successfully clustered plants in accordance with known evolutionary relationships, underscoring the significance of metabolite content as a taxonomic marker. Furthermore, the study discussed how metabolite content could serve as a predictor for nutritional and medicinal properties in plants, revealing previously unknown species-metabolite relationships.

In Fürtauer et al.’s study (2018), the emphasis was on understanding how abiotic stress influences the metabolic regulation of plants. The researchers utilized Arabidopsis wild-type plants and mutant lines with deficiencies in sucrose or starch metabolism, subjecting them to cold and high-light stress conditions. Through quantifying changes in the primary metabolome and proteome, they trained a machine-learning algorithm to classify mutant lines under control and stress conditions. This innovative approach identified a core module consisting of 23 proteins that reliably predicted combined temperature and high-light stress conditions. Importantly, 18 of these proteins were associated with protein-protein interactions, providing insights into the intricate biochemical regulation occurring in response to changing environmental conditions.

### 5.5 AI-assisted phenomics

DL techniques have proven remarkably effective in the realm of crop management, spanning various crops like rice, wheat, tomato, and potato. Li et al. (2020) pioneered a DL-based video detection system aimed at addressing plant diseases and pests in crops. Their primary goal was the swift identification of plant diseases and pests through comprehensive video analysis, utilizing advanced models such as Faster R-CNN and YOLO v3 for real-time video detection systems. The approach involved transforming videos into individual frames, analyzed using a Faster R-CNN framework for detection. Video-based evaluation metrics were introduced to assess detection quality, demonstrating that their custom backbone system outperformed existing systems in detecting untrained rice videos. Focusing on wheat stripe rust, a prevalent disease affecting wheat yields, Mi et al. (2020) introduced a novel DL network called C-DenseNet. This network incorporated the Convolutional Block Attention Module (CBAM) into a densely connected convolutional network (DenseNet), surpassing classical DenseNet and ResNet models in wheat stripe rust severity grading with a test accuracy of 97.99%.

Wang and Liu (2021) presented an early recognition method for tomato leaf spot using the MobileNetv2-YOLOv3 model. They enhanced recognition accuracy by introducing the GIoU bounding box regression loss function. This lightweight model demonstrated significant improvements in recognition performance compared to other models, achieving an F1 score of 94.13% under specific conditions. Addressing virus diseases in seed potatoes, Polder et al. (2019) proposed a hyperspectral imaging approach for field detection. They designed an imaging setup with a hyperspectral line-scan camera, training a convolutional neural network (CNN) on field data. The method achieved high precision and recall, showcasing its potential for real-world disease detection in potato crops. Chen and Yuan (2019) developed a deep-learning pipeline for localizing and counting agricultural pests in images. Their method integrated a convolutional neural network (CNN) and a region proposal network (RPN) with Non-Maximum Suppression (NMS) to remove overlapping detections. The model demonstrated high precision (0.93) with a low miss rate (0.10), showcasing its effectiveness in pest detection.

Feng et al. (2020) addressed plant defense against salinity stress using image processing and DL algorithms. They utilized high-throughput plant phenotyping technologies for non-destructive monitoring of plant traits. Employing hyperspectral imaging (HSI), the researchers assessed the phenotypes of 13 okra genotypes following salt treatment. Advanced plant and leaf segmentation techniques, coupled with DL algorithms, achieved outstanding results in accurately delineating plant and leaf structures. Salinity stress was found to have deleterious effects on okra’s physiological and biochemical processes, leading to significant alterations in spectral information. Leveraging this data, the study constructed predictive models for various traits, yielding promising results with correlation coefficients ranging from 0.588 to 0.835.

An overview of dedicated ML-based tools designed for addressing both abiotic and biotic stresses in plants are enlisted in **Table 2**. These specialized tools cater to specific types of stressors, offering a comprehensive resource for researchers and practitioners in the field of plant defense.

**Table 2:** AI-based tools for plant defense against abiotic and biotic stress.

Tool	Stressors	Features	Algorithm	Description	Website (Accessed on November 27, 2023)
ASRpro (Meher et al., 2022b)	Cold, drought, heat, light, oxidative and salt	autocross covariance & and K-mer composition	SVM	Identification of proteins associated with multiple abiotic stress in plants	https://iasri-sg.icar.gov.in/asrpro/
AsmiR (Pradhan et al., 2023b)	Drought, cold, salt and heat	pseudo-K-tuple nucleotide composition	SVM	Prediction of abiotic stress–specific miRNAs in plants	https://iasri-sg.icar.gov.in/asmir/
PLncPRO (Singh et al., 2017)	Abiotic	71 features were extracted using Framefinder (Slater, 2000) and BlastX (Altschul et al., 1997)	RF	Prediction of long abiotic stress-responsive long non-coding RNAs	http://ccbb.jnu.ac.in/plncpro/
ASRmiRNA (Meher et al., 2022a)	Abiotic	pseudo K-tuple nucleotide composition	SVM	Prediction of abiotic Stress-Responsive miRNA	http://cabgrid.res.in:8080/asrmirna
ASLncR (Pradhan et al., 2023a)	Abiotic	Kmer	SVM	Prediction of abiotic stress-responsive long non-coding RNAs	https://iasri-sg.icar.gov.in/aslncr/
DeepAProt (Ahmed et al., 2023)	Drought, cold, salinity and heat	46 features were extracted using bio-python package (Cock et al., 2009)	Long-short term memory	Identification and classification of abiotic stress protein sequences in cereals	http://login1.cabgrid.res.in:5500/
PredHSP (Kumar et al., 2016)	Heat	Dipeptide composition	SVM	Sequence-based prediction and classification of heat shock protein	http://14.139.227.92/mkumar/predhsp/index.html
iHSP-PseRAAC (Feng et al., 2013)	Heat	Spaced dipeptide composition	SVM	Identification of the heat shock protein families	http://lin.uestc.edu.cn/server/iHSP-PseRAAAC
ir-HSP (Meher et al., 2018)	Heat	pseudo amino acid composition	SVM	Classification of heat shock proteins sequences into one of the six heat shock proteins families	http://cabgrid.res.in:8080/ir-hsp
DeeperHSP (Min et al., 2021)	Heat	CNN	CNN	Identification of heat shock proteins	https://github.com/mswzeus/DeeperHSP
afpCOOL (Eslami et al., 2018)	Cold	Amino acid composition; evolutionary features	SVM	Prediction of anti-freeze proteins	–
AFP-Pred (Kandaswamy et al., 2011)	Cold	Overall composition of helix, stand and coil; Physicochemical properties, etc.	RF	Predicts antifreeze proteins from sequence-derived properties	https://www3.ntu.edu.sg/home/EPNSugan/index_files/AFP-Pred.htm
DRPPP (Pal et al., 2016)	Biotic	Protein sequence	SVM	Prediction of disease resistance proteins in plants	http://14.139.240.55/NGS/download.php
prPred (Wang et al., 2022)	Biotic	bidirectional long short-term memory	light gradient boosting	Prediction of plant resistance proteins	http://lab.malab.cn/soft/prPred-DRLF/
StackRPred (Chen et al., 2022)	Biotic	pairwise energy content of residues	Ensemble learning	Prediction of plant resistance proteins	–
ResCap (Kushwaha et al., 2021)	Biotic	Sequence compositional properties	SVM	Prediction of plant resistance gene	http://rescap.ltj.slu.se/ResCap/
EffectorP 3.0 (Sperschneider and Dodds, 2022)	Pathogen	Protein sequence features	Ensemble learning	Prediction of fungal and oomycetes effector proteins	https://effectorp.csiro.au/
InterSPPI (Yang et al., 2019)	Pathogens	Protein sequence features; network encoding	RF	Prediction of PPI between Arabidopsis and pathogens	http://systbio.cau.edu.cn/intersppi/index.php

Supervised ML and DL have been extensively applied in various plant biology studies, but there are situations where unsupervised and semi-supervised approaches are crucial. In the field of plant systems biology, unsupervised and semi-supervised learning algorithms play essential roles in diverse areas such as data clustering, dimensionality reduction (DR), visualization, gene regulatory network inference, cross-species prediction, and single-cell omics data analysis (Rai et al., 2019). PCA is widely used for DR and visualization of genotypic and multi-omics data (Yan et al., 2020). Hierarchical clustering is extensively employed for clustering genes with similar expression patterns in transcriptomic and proteomic research (Xu et al., 2012; Klepikova et al., 2016). Algorithms like t-sne and optics contribute significantly to the analysis of genotypic data, enhancing visualization of large-scale maize hybrid populations’ structures (Yan et al., 2021). Non-negative matrix factorization (NMF) proves valuable in breaking down expression matrices with thousands of genes into a small number of metagenes in Arabidopsis and maize (Wilson et al., 2012; Ma et al., 2022). The multifactor dimensionality reduction (MDR) algorithm is employed for identifying multiple pairwise epistatic effects and gene–environment interactions affecting agronomic and quality traits in rice and barley (Xu et al., 2015; Xu et al., 2018).

Semi-supervised and transfer learning strategies have emerged to overcome the scarcity of annotated genes and pathways in plants. Transfer learning was employed to predict specialized/general metabolism-related genes in *Solanum lycopersicum* (tomato) by leveraging well-annotated Arabidopsis genes (Moore et al., 2020). Another innovative approach, ‘evolutionarily informed machine learning,’ used an xgboost model trained on transcriptomic data in Arabidopsis to predict nitrogen-use efficiency (NUE) and related genes in maize (Cheng et al., 2021). With the advent of single-cell sequencing technology, challenges arise due to higher dimensionality and complexity (Bobrovskikh et al., 2021). Advanced algorithms like t-sne, umap, magic, phate, Saucie, and Beeline have been proposed to address these challenges and are prevalent in both human and plant studies (Van Dijk et al., 2018; Amodio et al., 2019; Becht et al., 2019; Pratapa et al., 2020; Wu and Zhang, 2020; Marand et al., 2021).

## 6 Challenges

The rapid advancements in biological data generation and ML development have opened up significant possibilities for unraveling complex biological information. However, integrating ML into plant molecular studies poses notable challenges. ML approaches, similar to traditional plant molecular methods, are highly context-specific, underscoring the importance of meticulous experimental design. It’s essential to recognize that while ML aims to create predictive models, each ML algorithm comes with distinct strengths and weaknesses, influencing predictive efficiency under specific conditions. Consequently, an ML model crafted for one dataset may struggle to generalize well to others due to inherent biological and technical variations.

The abundance of omics datasets provides a treasure trove of information. However, a notable portion of these datasets is marked by characteristics such as noise and sparsity. This poses a substantial challenge when it comes to accurately identifying biological features, especially during the integration of various omics data sources (Joyce and Palsson, 2006). The challenge of imbalanced datasets, where sample sizes vary across categories, is pervasive in ML. Researchers address this through resampling strategies such as oversampling and undersampling (Maimon and Rokach, 2005). For instance, Li et al. (2021) employed the synthetic minority oversampling technique (SMOTE) to bolster the representation of minority cases, a crucial step in the identification of effector proteins. The presence of noise and sparsity in these datasets introduces uncertainty and complexity, potentially hindering the identification of meaningful patterns or features within the biological data. Researchers must grapple with the task of distinguishing genuine biological signals from the background noise, emphasizing the need for robust analytical approaches to ensure the reliability of findings. Additionally, the issue of overfitting looms prominently, particularly in the domain of DL. Overfitting occurs when a model becomes overly tailored to the intricacies of a specific dataset to the extent that it struggles to generalize well to new, unseen data. This phenomenon can compromise the model’s predictive capabilities and hinder its applicability to real-world scenarios. In addressing this concern, techniques like dropout have been employed (Scholz et al., 2004).

Dropout is a regularization technique that involves randomly “dropping out” or deactivating a subset of neurons during the training of a neural network. By doing so, dropout helps prevent the neural network from becoming overly reliant on specific features or relationships present in the training data, thereby enhancing its ability to generalize to new and unseen data. This technique acts as a safeguard against overfitting, promoting a more robust and adaptable model. Various factors, including data preprocessing, user-defined parameters, and domain knowledge, significantly influence the effectiveness of ML models. ML practitioners play a pivotal role in decision-making throughout the process, underscoring the importance of incorporating prior knowledge and domain expertise to unveil meaningful patterns.

Dealing with big data characteristics in plant system biology studies, encompassing volume, variety, veracity, value, and velocity, presents its own set of challenges. ML methods must adapt to handle multi-omics data, considering the unique insights each omics layer provides. Challenges include addressing high-dimensional data issues such as sparsity, multicollinearity, and overfitting, necessitating tailored methods and collaborative efforts in data integration.

Interpreting complex models, particularly in advanced ML approaches like DL, remains challenging due to their ‘black box’ nature. Researchers often prioritize understanding the biological significance of a predictive model over its accuracy, requiring careful processing and correlation with existing biological knowledge.

Despite these challenges, the studies discussed in this context represent success stories in the application of AI in plant omics. To fully harness the potential of AI, robust and scalable algorithms that uncover meaningful biological insights are crucial. Ensuring accuracy and reliability through experimental validation is essential for translating computational findings into practical applications. Bridging collaboration gaps between omics researchers, data scientists, and agricultural experts is vital for realizing the full potential of AI in plant defense and practical applications.

## 7 Future directions

Integration of AI and Omics Data in Early Disease Detection: One promising future direction is the seamless integration of AI with various omics data (genomics, transcriptomics, proteomics, and metabolomics) to enable the early detection of plant diseases. Advanced AI algorithms can analyze multi-dimensional omics datasets to identify subtle changes in plant molecular profiles associated with disease onset, even before visible symptoms emerge.

High-Throughput Phenotyping with AI-Omics Fusion: Combining AI-assisted omics techniques with high-throughput phenotyping methods offers an exciting avenue. This fusion can enable real-time monitoring of plant health by linking molecular responses to observable phenotypic traits. The integration of phenomics data into AI-driven analysis pipelines enhances our understanding of plant defense mechanisms.

Predictive Modeling of Disease Dynamics: Leveraging AI and omics data, predictive modeling can be developed to forecast disease dynamics in plant populations. ML and DL models can factor in genetic, molecular, and environmental variables to predict disease outbreaks and assess the impact of preventive measures.

Customized Crop Breeding: Future research may focus on using AI-assisted omics techniques to tailor crop breeding programs for enhanced disease resistance. By pinpointing specific genetic markers and pathways associated with resistance, breeders can design crops with improved defense mechanisms.

AI-Guided Sustainable Disease Management: AI can assist in optimizing disease management strategies. Integrating AI-powered recommendations with omics data allows for precision application of pesticides, reducing environmental impact and lowering costs while effectively controlling plant pathogens.

Addressing Combined Stressors: With climate change and evolving agricultural practices, plants often face the challenge of multiple stressors simultaneously. Future research should focus on understanding how plants respond to combined biotic and abiotic stress, as this represents a significant real-world scenario. AI-assisted omics techniques can play a pivotal role in unraveling the intricate interactions between different stress factors and their cumulative effects on plant defense mechanisms. This knowledge is essential for developing holistic and resilient strategies to protect crops in complex stress environments, ensuring sustainable food production.

Integration of Remote Sensing Data with AI-Omics Fusion: The integration of remote sensing technology with AI-assisted omics techniques offers a powerful approach to monitor and mitigate plant stress. Remote sensing provides valuable spatial and temporal data on plant health, stress factors, and environmental conditions. By merging remote sensing data with omics information, researchers can gain a comprehensive understanding of the interplay between genetic responses and environmental stressors. This integrated approach enables more precise and timely interventions to enhance plant defense and reduce crop losses.

## 8 Conclusion

In conclusion, this comprehensive review explores the landscape of omics studies in plant defense against biotic and abiotic stress, and the transformative role of ML techniques in various omics domains. From genomics to metabolomics, AI-assisted techniques showcase their prowess in extracting meaningful insights from expansive datasets, surpassing traditional methods. While emphasizing the advantages of ML, the review also addresses the challenges associated with its implementation in plant omics, paving the way for future developments. Progress in computational frameworks facilitates the seamless application of modern methods. With the increasing volume of plant sequencing data, ML emerges as a catalyst in accelerating various facets of plant genomic research. This includes pinpointing genes associated with resistance against biotic and abiotic stress, as well as enhancing our comprehension of gene regulation mechanisms. These strides are poised to aid agricultural researchers in enhancing crop yield and quality, fostering improved resilience to biotic and abiotic stressors.

## Author contributions

SM: Writing – original draft. DS: Writing – original draft. HC: Writing – original draft. SS: Writing – review & editing. RD: Writing – review & editing. GJ: Writing – review & editing. SA: Funding acquisition, Writing – review & editing.
